# Supplementary material for: Modeling glioblastoma heterogeneity as a dynamic network of cell states
Source: Mol Syst Biol. 2021 Sep 16;17(9):e10105. doi: 10.15252/msb.202010105 (PMC8444284; doi:10.15252/msb.202010105)
Supplement: Supplementary file 5 — Source Data for Figure 3 [file MSB-17-e10105-s001.zip › Figure3A_sourcedata/GSEA_3065/nosigdb_state4.GseaPreranked.1622718430994/DALMO_BARCODENUMBER_UNPUB.html]

Details for gene set DALMO\_BARCODENUMBER\_UNPUB[GSEA]

|  || Dataset | state4 |
| Phenotype | NoPhenotypeAvailable |
| Upregulated in class | na\_neg |
| GeneSet | DALMO\_BARCODENUMBER\_UNPUB |
| Enrichment Score (ES) | -0.7146898 |
| Normalized Enrichment Score (NES) | -2.7800114 |
| Nominal p-value | 0.0 |
| FDR q-value | 0.0 |
| FWER p-Value | 0.0 |
Table: GSEA Results Summary

  

Fig 1: Enrichment plot: DALMO\_BARCODENUMBER\_UNPUB      
 Profile of the Running ES Score & Positions of GeneSet Members on the Rank Ordered List

  

| PROBE | GENE SYMBOL | GENE\_TITLE | RANK IN GENE LIST | RANK METRIC SCORE | RUNNING ES | CORE ENRICHMENT || 1 | BEX1 |  |  | 11 | 0.473 | 0.0076 | No |
| 2 | NLRP1 |  |  | 16 | 0.425 | 0.0153 | No |
| 3 | SPARCL1 |  |  | 17 | 0.416 | 0.0236 | No |
| 4 | ZMAT3 |  |  | 34 | 0.338 | 0.0276 | No |
| 5 | NEAT1 |  |  | 41 | 0.315 | 0.0329 | No |
| 6 | C9orf16 |  |  | 45 | 0.306 | 0.0384 | No |
| 7 | ATP6V1G1 |  |  | 47 | 0.303 | 0.0443 | No |
| 8 | PHPT1 |  |  | 63 | 0.282 | 0.0474 | No |
| 9 | DKK3 |  |  | 66 | 0.281 | 0.0526 | No |
| 10 | PLEKHB1 |  |  | 82 | 0.262 | 0.0554 | No |
| 11 | APLP1 |  |  | 102 | 0.246 | 0.0571 | No |
| 12 | CYSTM1 |  |  | 108 | 0.244 | 0.0611 | No |
| 13 | VGLL4 |  |  | 116 | 0.241 | 0.0648 | No |
| 14 | IFI27L2 |  |  | 133 | 0.229 | 0.0667 | No |
| 15 | RPS27L |  |  | 149 | 0.223 | 0.0686 | No |
| 16 | PCSK1N |  |  | 172 | 0.212 | 0.0692 | No |
| 17 | ZBTB20 |  |  | 275 | 0.180 | 0.0560 | No |
| 18 | FAM210B |  |  | 311 | 0.172 | 0.0536 | No |
| 19 | CDKN1A |  |  | 319 | 0.171 | 0.0559 | No |
| 20 | TP53I3 |  |  | 353 | 0.164 | 0.0537 | No |
| 21 | GDPD2 |  |  | 477 | 0.141 | 0.0362 | No |
| 22 | LAMB2 |  |  | 510 | 0.135 | 0.0336 | No |
| 23 | PSAP |  |  | 596 | 0.126 | 0.0221 | No |
| 24 | PLTP |  |  | 625 | 0.123 | 0.0199 | No |
| 25 | GSTM3 |  |  | 645 | 0.121 | 0.0192 | No |
| 26 | BTG2 |  |  | 689 | 0.116 | 0.0144 | No |
| 27 | TCEAL7 |  |  | 705 | 0.113 | 0.0142 | No |
| 28 | CHPF |  |  | 706 | 0.113 | 0.0164 | No |
| 29 | S100A10 |  |  | 812 | 0.103 | 0.0012 | No |
| 30 | MMP2 |  |  | 813 | 0.103 | 0.0032 | No |
| 31 | YPEL3 |  |  | 822 | 0.102 | 0.0039 | No |
| 32 | RRM2B |  |  | 932 | 0.093 | -0.0122 | No |
| 33 | NUDT14 |  |  | 993 | 0.089 | -0.0203 | No |
| 34 | HTRA1 |  |  | 1008 | 0.088 | -0.0209 | No |
| 35 | AKIRIN1 |  |  | 1137 | 0.079 | -0.0404 | No |
| 36 | KIF3C |  |  | 1152 | 0.079 | -0.0411 | No |
| 37 | RPL35 |  |  | 1187 | 0.077 | -0.0452 | No |
| 38 | RAMP1 |  |  | 1283 | 0.072 | -0.0594 | No |
| 39 | SEZ6L2 |  |  | 1298 | 0.071 | -0.0603 | No |
| 40 | MDM2 |  |  | 1450 | 0.064 | -0.0839 | No |
| 41 | TRIM22 |  |  | 1640 | 0.057 | -0.1139 | No |
| 42 | PCDHB9 |  |  | 1724 | 0.055 | -0.1265 | No |
| 43 | SEZ6L |  |  | 1768 | 0.053 | -0.1325 | No |
| 44 | IP6K2 |  |  | 1813 | 0.052 | -0.1387 | No |
| 45 | LONRF2 |  |  | 1824 | 0.052 | -0.1393 | No |
| 46 | BLOC1S2 |  |  | 1883 | 0.050 | -0.1479 | No |
| 47 | DDR1 |  |  | 1941 | 0.048 | -0.1563 | No |
| 48 | PHLDA3 |  |  | 2311 | 0.039 | -0.2163 | No |
| 49 | PROS1 |  |  | 2312 | 0.039 | -0.2155 | No |
| 50 | SNHG8 |  |  | 2468 | 0.036 | -0.2403 | No |
| 51 | CHST7 |  |  | 2773 | 0.029 | -0.2898 | No |
| 52 | ST8SIA1 |  |  | 2921 | 0.026 | -0.3135 | No |
| 53 | LRP1 |  |  | 2940 | 0.026 | -0.3159 | No |
| 54 | MAN2B2 |  |  | 3186 | 0.021 | -0.3559 | No |
| 55 | RETSAT |  |  | 3588 | 0.009 | -0.4217 | No |
| 56 | TMEM59L |  |  | 3617 | 0.008 | -0.4261 | No |
| 57 | IGSF8 |  |  | 3770 | 0.002 | -0.4511 | No |
| 58 | CTSF |  |  | 3820 | -0.000 | -0.4592 | No |
| 59 | GAA |  |  | 4119 | -0.017 | -0.5079 | No |
| 60 | ABCA5 |  |  | 4189 | -0.021 | -0.5189 | No |
| 61 | IDS |  |  | 4199 | -0.022 | -0.5199 | No |
| 62 | XRCC6 |  |  | 4437 | -0.039 | -0.5582 | No |
| 63 | SNRPF |  |  | 4498 | -0.044 | -0.5671 | No |
| 64 | ENY2 |  |  | 4593 | -0.051 | -0.5816 | No |
| 65 | TM2D1 |  |  | 4708 | -0.060 | -0.5992 | No |
| 66 | POLD2 |  |  | 4714 | -0.061 | -0.5988 | No |
| 67 | HOOK2 |  |  | 4885 | -0.074 | -0.6253 | No |
| 68 | CHD9 |  |  | 4909 | -0.075 | -0.6276 | No |
| 69 | VPS13C |  |  | 4912 | -0.076 | -0.6265 | No |
| 70 | PLXNB2 |  |  | 4962 | -0.080 | -0.6329 | No |
| 71 | PCDH9 |  |  | 4972 | -0.080 | -0.6328 | No |
| 72 | SKA3 |  |  | 5136 | -0.095 | -0.6578 | No |
| 73 | PLK4 |  |  | 5210 | -0.102 | -0.6678 | No |
| 74 | GADD45A |  |  | 5297 | -0.109 | -0.6798 | No |
| 75 | ITGAV |  |  | 5309 | -0.110 | -0.6794 | No |
| 76 | THOC3 |  |  | 5312 | -0.110 | -0.6775 | No |
| 77 | GPC1 |  |  | 5348 | -0.113 | -0.6811 | No |
| 78 | H2AFV |  |  | 5381 | -0.117 | -0.6840 | No |
| 79 | LRR1 |  |  | 5459 | -0.126 | -0.6942 | No |
| 80 | COL18A1 |  |  | 5543 | -0.135 | -0.7052 | No |
| 81 | HPRT1 |  |  | 5554 | -0.136 | -0.7041 | No |
| 82 | SPDL1 |  |  | 5560 | -0.137 | -0.7022 | No |
| 83 | HMGB3 |  |  | 5584 | -0.142 | -0.7032 | No |
| 84 | CENPW |  |  | 5585 | -0.142 | -0.7004 | No |
| 85 | NCAPH |  |  | 5595 | -0.144 | -0.6990 | No |
| 86 | KIFC1 |  |  | 5645 | -0.152 | -0.7041 | No |
| 87 | DAZAP1 |  |  | 5679 | -0.156 | -0.7064 | No |
| 88 | PARPBP |  |  | 5682 | -0.157 | -0.7036 | No |
| 89 | CCT8 |  |  | 5704 | -0.161 | -0.7039 | No |
| 90 | ITGA7 |  |  | 5715 | -0.163 | -0.7023 | No |
| 91 | KIF18B |  |  | 5760 | -0.174 | -0.7061 | No |
| 92 | HNRNPM |  |  | 5797 | -0.182 | -0.7084 | No |
| 93 | PLK1 |  |  | 5836 | -0.192 | -0.7109 | Yes |
| 94 | FANCD2 |  |  | 5849 | -0.195 | -0.7090 | Yes |
| 95 | PAICS |  |  | 5858 | -0.196 | -0.7064 | Yes |
| 96 | IGFBP7 |  |  | 5868 | -0.199 | -0.7040 | Yes |
| 97 | ANP32B |  |  | 5890 | -0.204 | -0.7034 | Yes |
| 98 | DIAPH3 |  |  | 5893 | -0.205 | -0.6997 | Yes |
| 99 | PTGES3 |  |  | 5912 | -0.210 | -0.6985 | Yes |
| 100 | PRR11 |  |  | 5926 | -0.214 | -0.6964 | Yes |
| 101 | S100A6 |  |  | 5930 | -0.215 | -0.6926 | Yes |
| 102 | ACAT2 |  |  | 5931 | -0.215 | -0.6883 | Yes |
| 103 | LSM5 |  |  | 5932 | -0.216 | -0.6841 | Yes |
| 104 | SMS |  |  | 5940 | -0.218 | -0.6809 | Yes |
| 105 | RACGAP1 |  |  | 5953 | -0.223 | -0.6784 | Yes |
| 106 | CIP2A |  |  | 5956 | -0.225 | -0.6743 | Yes |
| 107 | CENPA |  |  | 5960 | -0.226 | -0.6703 | Yes |
| 108 | ATP6V0B |  |  | 5961 | -0.226 | -0.6659 | Yes |
| 109 | PCMT1 |  |  | 5973 | -0.228 | -0.6631 | Yes |
| 110 | TROAP |  |  | 5983 | -0.232 | -0.6600 | Yes |
| 111 | MZT1 |  |  | 5989 | -0.235 | -0.6562 | Yes |
| 112 | KIF2C |  |  | 5996 | -0.237 | -0.6525 | Yes |
| 113 | SPC24 |  |  | 6000 | -0.238 | -0.6483 | Yes |
| 114 | POC1A |  |  | 6010 | -0.241 | -0.6450 | Yes |
| 115 | BUB1 |  |  | 6045 | -0.266 | -0.6453 | Yes |
| 116 | HMGB1 |  |  | 6047 | -0.267 | -0.6402 | Yes |
| 117 | PGRMC1 |  |  | 6051 | -0.270 | -0.6353 | Yes |
| 118 | HJURP |  |  | 6055 | -0.271 | -0.6304 | Yes |
| 119 | LDHA |  |  | 6057 | -0.272 | -0.6252 | Yes |
| 120 | GRIA2 |  |  | 6065 | -0.277 | -0.6208 | Yes |
| 121 | ARHGAP11A |  |  | 6067 | -0.277 | -0.6155 | Yes |
| 122 | FOXM1 |  |  | 6070 | -0.279 | -0.6103 | Yes |
| 123 | SPC25 |  |  | 6072 | -0.279 | -0.6050 | Yes |
| 124 | CENPN |  |  | 6090 | -0.291 | -0.6020 | Yes |
| 125 | DEK |  |  | 6092 | -0.292 | -0.5964 | Yes |
| 126 | NUF2 |  |  | 6093 | -0.293 | -0.5906 | Yes |
| 127 | NCAPG2 |  |  | 6094 | -0.294 | -0.5847 | Yes |
| 128 | CDCA5 |  |  | 6099 | -0.296 | -0.5795 | Yes |
| 129 | CCNA2 |  |  | 6100 | -0.297 | -0.5737 | Yes |
| 130 | PIF1 |  |  | 6106 | -0.301 | -0.5685 | Yes |
| 131 | CKAP2L |  |  | 6108 | -0.303 | -0.5627 | Yes |
| 132 | KIF11 |  |  | 6109 | -0.304 | -0.5566 | Yes |
| 133 | COL9A3 |  |  | 6111 | -0.305 | -0.5507 | Yes |
| 134 | PIMREG |  |  | 6115 | -0.310 | -0.5451 | Yes |
| 135 | CCT5 |  |  | 6116 | -0.312 | -0.5389 | Yes |
| 136 | MIS18BP1 |  |  | 6122 | -0.316 | -0.5335 | Yes |
| 137 | SRSF2 |  |  | 6128 | -0.321 | -0.5279 | Yes |
| 138 | PHF19 |  |  | 6129 | -0.322 | -0.5215 | Yes |
| 139 | HMGA1 |  |  | 6132 | -0.324 | -0.5154 | Yes |
| 140 | CDCA2 |  |  | 6135 | -0.326 | -0.5093 | Yes |
| 141 | TACC3 |  |  | 6138 | -0.328 | -0.5031 | Yes |
| 142 | CCNB2 |  |  | 6140 | -0.329 | -0.4968 | Yes |
| 143 | AURKB |  |  | 6143 | -0.332 | -0.4905 | Yes |
| 144 | TK1 |  |  | 6144 | -0.334 | -0.4839 | Yes |
| 145 | CDC20 |  |  | 6154 | -0.344 | -0.4786 | Yes |
| 146 | DLGAP5 |  |  | 6156 | -0.345 | -0.4719 | Yes |
| 147 | KIF15 |  |  | 6158 | -0.346 | -0.4652 | Yes |
| 148 | CDKN3 |  |  | 6160 | -0.349 | -0.4584 | Yes |
| 149 | KIF23 |  |  | 6162 | -0.351 | -0.4516 | Yes |
| 150 | HMGN2 |  |  | 6163 | -0.351 | -0.4447 | Yes |
| 151 | KIF4A |  |  | 6164 | -0.355 | -0.4377 | Yes |
| 152 | CDCA3 |  |  | 6165 | -0.355 | -0.4306 | Yes |
| 153 | ANP32E |  |  | 6168 | -0.357 | -0.4239 | Yes |
| 154 | ODC1 |  |  | 6175 | -0.377 | -0.4174 | Yes |
| 155 | CENPM |  |  | 6179 | -0.386 | -0.4102 | Yes |
| 156 | SGO1 |  |  | 6188 | -0.404 | -0.4036 | Yes |
| 157 | SGO2 |  |  | 6192 | -0.413 | -0.3959 | Yes |
| 158 | TUBB4B |  |  | 6196 | -0.431 | -0.3878 | Yes |
| 159 | RRM2 |  |  | 6199 | -0.438 | -0.3795 | Yes |
| 160 | DHFR |  |  | 6200 | -0.441 | -0.3707 | Yes |
| 161 | ESCO2 |  |  | 6206 | -0.463 | -0.3624 | Yes |
| 162 | PBK |  |  | 6207 | -0.465 | -0.3532 | Yes |
| 163 | BIRC5 |  |  | 6214 | -0.484 | -0.3445 | Yes |
| 164 | ANLN |  |  | 6215 | -0.484 | -0.3349 | Yes |
| 165 | HIST1H1A |  |  | 6216 | -0.487 | -0.3253 | Yes |
| 166 | GTSE1 |  |  | 6217 | -0.490 | -0.3156 | Yes |
| 167 | NUSAP1 |  |  | 6223 | -0.507 | -0.3064 | Yes |
| 168 | CENPE |  |  | 6226 | -0.511 | -0.2966 | Yes |
| 169 | NCAPG |  |  | 6227 | -0.513 | -0.2864 | Yes |
| 170 | SMC2 |  |  | 6229 | -0.518 | -0.2763 | Yes |
| 171 | CKS1B |  |  | 6234 | -0.523 | -0.2666 | Yes |
| 172 | KPNA2 |  |  | 6236 | -0.543 | -0.2560 | Yes |
| 173 | H2AFX |  |  | 6237 | -0.576 | -0.2445 | Yes |
| 174 | CKS2 |  |  | 6239 | -0.585 | -0.2331 | Yes |
| 175 | UBE2T |  |  | 6240 | -0.593 | -0.2214 | Yes |
| 176 | CCNB1 |  |  | 6242 | -0.606 | -0.2095 | Yes |
| 177 | PTTG1 |  |  | 6245 | -0.622 | -0.1975 | Yes |
| 178 | CDK1 |  |  | 6246 | -0.627 | -0.1851 | Yes |
| 179 | MAD2L1 |  |  | 6247 | -0.628 | -0.1726 | Yes |
| 180 | HIST1H1B |  |  | 6249 | -0.646 | -0.1600 | Yes |
| 181 | H2AFZ |  |  | 6250 | -0.651 | -0.1471 | Yes |
| 182 | TPX2 |  |  | 6253 | -0.667 | -0.1342 | Yes |
| 183 | ASPM |  |  | 6254 | -0.680 | -0.1208 | Yes |
| 184 | UBE2C |  |  | 6255 | -0.750 | -0.1059 | Yes |
| 185 | HMGB2 |  |  | 6258 | -0.766 | -0.0910 | Yes |
| 186 | PCLAF |  |  | 6259 | -0.790 | -0.0754 | Yes |
| 187 | TOP2A |  |  | 6260 | -0.814 | -0.0592 | Yes |
| 188 | MKI67 |  |  | 6261 | -0.882 | -0.0417 | Yes |
| 189 | HIST1H4C |  |  | 6262 | -1.038 | -0.0212 | Yes |
| 190 | CENPF |  |  | 6263 | -1.068 | -0.0000 | Yes |
Table: GSEA details [plain text format]

  

Fig 2: DALMO\_BARCODENUMBER\_UNPUB: Random ES distribution      
 Gene set null distribution of ES for **DALMO\_BARCODENUMBER\_UNPUB**
